# On the Influence of Names, and on the State of the Medical Profession

**Published:** 1817-10

**Authors:** Andrew Blake

**Affiliations:** Member of the Royal College of Surgeons, London.


*For the London Medical and Physical Journal.*

### *On the Influence of Names, and on the State of the Medical Profession;* by ANDREW BLAKE, M.D. Member of the Royal College of Surgeons, London.

"Names are the stumbling-blocks of all improvement, and their influence is always directly in proportion to our want of real knowledge."

IT is devoutly to be prayed for by this country at large, that legislative interference may adopt some coercive regulation, restricting the future practice of midwifery to those who, with due pretensions, amply qualify themselves before some professional tribunal for that purpose. It is undoubtedly true, and cannot be sufficiently lamented, that there are an infinite number of worthless pretenders in this country, who, neither by education, talent, nor experience, are adapted to the vital exercise of that momentous duty, who, from the veriest menial capacities, at once assume an office, the eminent functions of which they are really unacquainted with, are daring and obtrusive from the thirst of profit and mistrusted encouragement, and hardened to every sense of conscientious feeling from long inveteracy in the total disavowal of it.

I am urged by no personal animosity or unworthy impulse, and should not, perhaps, here have thus expressed myself, had I not recently been consulted in a case at a moment when all consultation was useless, and in which, I affirm it unhesitatingly, a lady lost her valuable life, a father and children an incalculable treasure, by the perverse and criminal ignorance of an attending man-midwife.

This man was proverbially illiberal to his professional neighbours, he professed all that forward assumption which characterizes little talent, bold conceit, and regardless attention of danger; upon every occasion, however intricate, he so valued his own services, as to hold those of others, when sought for, as contemptible and unnecessary; he had arrived at those years which inspire confidence and carry with them the unerring conviction of supreme infallibility, and he dealt largely in happiness or misery according to the bias of the moment, and the adequateness of nature in her struggles to cast off sickness and disease.

In this instance the family were particularly solicitous, but, by his repeated assurances of safety, they were lulled into a negative satisfaction for the time: the fatal progress, however, of the malady was rapid, and, the general appearance of the patient indicating too plainly to the by-standers

 the the speedy closing of the scene, I was, without further importunity, sent for, being then accidentally in the neighbourhood. On my arrival, the whole appearances foretold immediate dissolution, a ghastly countenance, laborious respiration, imperceptible pulse, and rapid hæmorrhage without pain. After being in the apartment a few minutes, and while making some necessary enquiries, she expired, or rather bled to death. I was then addressed with much hauteur and pedantic all-sufficiency by this gentleman, in the following words:—

"This, Sir, was a placental presentation, in which I deemed it requisite to puncture the amnion tumour to expedite the parturient endeavours, and to cessate thereby the hæmorrhagic effusion by the progressive pressure of the fœtal head; my disappointment in these earnest expectations compelled me to the operation of plugging the vagina, as the only resource left for saving my patient, and you have just witnessed this was ineffectual.—This, Sir, is one of those melancholy cases which frustrate the best endeavours of the obstetric art."

I never attend a patient in a dangerous illness that I do not sincerely pray for; and it is to me an anomaly in human nature, that men can be notoriously culpable, made acquainted with their error, and yet show no anguish of sensation. I sincerely hope there are not many families exposed to the same unhappy probabilities; but, could some be made sensible of the professional talent they encourage and confide in, they would involuntarily shudder at the recollection of averted perils, and tremble fearfully for those whose misguided credulity may expose to similar misfortunes. Man will attempt any thing for the mercenary compensation of lucre, when he unrepentingly trifles with the lives of the most lovely individuals, to the ruin of domestic joys, the irreparable injury of society, and the disgrace of an enlightened country. It is such men that compel the modest and accomplished scholar to linger in heart-breaking obscurity, on the bitter morsel of poverty and hopelessness; it is such men that have driven others to seek a foreign home, with their all, the unambitious hope of barely living; while some, not possessing so much virtue in difficulties, were lost in despair, or perished in the commission of crime. True learning is modest, is diffident; and, unless some good friend to talent kindly fosters it with becoming encouragement and hope, it relaxes, insupportably burthened with its own unmerited obscurity, into slothfulness, imbecility, and despair; and this state is not a little embittered by daily witnessing the goading injustice of worthlessness in prosperity.

 It

It is, indeed, time, that the condition of our profession should be ameliorated, and, during the present suspension of parliamentary duties, it will be well some nervous resolutions were adopted and brought forward to attract the attention of authority; in the mean time, it is our bounden duty, individually and collectively, to urge its best interests in every possible way,—for it must be the first hope of him who loves his profession, to see it emerge from its present degrading obscurity, and rise at least to a deserved rank in the higher department of philosophy; to see its members dignified with their original honours, and their industry remunerated with the emoluments its sacred vocation claims.

The progress of medicine in this country is thwarted and discouraged by public connivance at unrestricted quackery, and the open practices of unlicensed pretenders to the art; its welfare is scarcely noticed by the legislature or patronized by the prerogative, while the members of the sister professions are dignified with the highest honours of the state; it is, as a science, exclusively and unjustly debarred from every prospect of aggrandisement. With this want of necessary stimulus to learning, or rather with this goading and oppressive injustice constantly before us, it would not have been very questionable had medicine retrograded, instead of having struggled to uphold its character amidst the scale of arts and sciences; but this is attributable entirely to the characteristic perseverance of an Englishman, that in whatever he attempts he accomplishes; and it is to that inherent impulsive emulation alone we ascribe the present state of anatomy, the grand foundation on which all superstructure rests.

It is not on a corrupt and untenable foundation that medical philosophy can pave the way to well-merited distinction, and receive the unlimited homage of society; it must be on just and firm principles, a government of immutable laws and logical reasoning, divested of all vague and hypothetical delusion. But, to establish pathology on an infallible and unerring base, must be a gifted birth-right, emanating from the decrees of Divine Providence, to him who is to illustrate the principles of life, the laws on which vitality depend, and the government directing all their functions: till then, we must, from these unknown peculiarities of existence, humbly content ourselves with the labours of observation and reflection, and, aided by the power of anatomical research, to part the misty envelope which overshadows the talents of the most eminent.

If physicians of learning and ability will ever content themselves with tamely relying on the dull and hacknied proverbs of their forefathers,—if they will not think, judge, and and act for themselves, what advancement can the profession expect from the labours of experience? I had rather bear the lash of animadversion, in the hope it might lead to some useful controversy, and be productive thereby of advantage to my profession by eliciting truth, than contemptibly whirl in the beaten track of my predecessors, by whose antiquated proselytes in pathology each revered aphorism is carefully cherished with a superstitious conviction of its practical utility, uniting the most unbending disbelief in the frequency of its mischievous failure.

Not but that I venerate the memory of our great masters, their practical doctrines laid the solid foundation of a curative system, that stands the incontrovertible test of approving times; but with them, it is to be regretted, has been blended much erroneous matter, and, promulgated by names which unfortunately carried with it the stubborn belief of supreme efficacy, it has proved an effectual barrier to the gradual advancement of medical literature. No errors can have such fatal tendency, none so inveterate, as those which are taught and willed to children; they are a contaminating stream, from which indefinite numbers may pollute themselves, and, when sanctioned by authority and consecrated by adoption, it is to be expected they will imbibe the same innate assurances of their specific sovereignty over all verified improvements, because forsooth they are modern, and therefore chimerical innovations.

Thus, in this simple instance, because Hippocrates has said, that beer and milk are alike hurtful in fever, the generality of authors have prejudged them so, and, with this absurd belief, they scrupulously lay the most profound stress on their denial to the sick, although they never witnessed any ill effects from either: beer, particularly porter, is one of the best diffusible stimulants we know in the last stage of fever, and milk is not only a grateful beverage, but appropriate diet also. Such is the favourable prejudice of early doctrines; they are the indolent dogmas which constitute the empirical routine of those who walk by rule, and practise strictly conformably to nosological definition.

The daily practice of a physician must exemplify the mournful insufficiency of all human endeavour in the alleviation of mortal suffering; and, when we reflect, the future happiness or misery of afflicted individuals who press around the bed of sickness, await the feeble result of a wavering determination, that their sole hope unconsciously depends on an erring, or, perhaps a faulty, judgment; when we see the ineffectual struggle, the last gleam of life preparing to fade before our view, we cannot but regret with pain how imbecile is the pompous pedantry of learning, and acknowledge, with humility, how futile is our assumptive title to philosophy. For the doctrines, therefore, which are handed down to us with such implicit faith, we ought not to be so regardful of names, either individually or collectively, as to be fearfully deterred from instituting a free investigation into their hypothesis, or bettered in our judgment as to their fallacy or virtue; and the remedy which may appear less objectionable ought to be urged by the convicted result of repeated cases,—a disinterested but emulous desire to enhance the welfare of the profession, and not from the vain and ambitious propensity of committing to the press, for the sake of an ephemeral notoriety, what must appear wondrous to others because unintelligible to most. We are not to be deterred from experimental enquiry, which, as well as the constant observance of facts, is requisite to explain the *modus operandi* of medicines we are in the daily habit of prescribing, without which, we can neither practise conscientiously or with a happy result.

We ought also, on so important a subject as health, to confine our language to unadorned perspicuity, in lieu of elegancy of diction. As an author, I shall always approve of the scientific man who openly opposes any opinions I may now or hereafter espouse, if, with a freedom of latitude, he unites a liberality of sentiment and the intentions of the gentleman.

*August* 1817.

